# Prognostic significance of positive peritoneal cytology in resectable pancreatic cancer: a systemic review and meta-analysis

**DOI:** 10.18632/oncotarget.14745

**Published:** 2017-01-19

**Authors:** Feng Cao, Jia Li, Ang Li, Fei Li

**Affiliations:** ^1^ Department of General Surgery, Xuanwu Hospital, Capital Medical University, Beijing 100053, PR China

**Keywords:** pancreatic cancer, surgery, cytology, overall survival, meta-analysis

## Abstract

Although peritoneal cytology has been used to determine pancreatic cancer staging for more than three decades, its prognostic significance in potentially resectable pancreatic cancer is inconclusive. We therefore conducted this meta-analysis to investigate the impact of peritoneal cytology status on the clinicopathological features and survival outcomes in potentially resectable pancreatic cancer. Ten studies were identified for this meta-analysis after searching the PubMed, Web of Science and China National Knowledge Infrastructure (CNKI) electronic databases. Our results showed that positive peritoneal cytology was associated with tumor size (OR 11.65, *P* = 0.001), tumor location (OR 0.37, *P* = 0.000), serosal invasion (OR 3.89, *P* = 0.000), portal vein invasion (OR 1.82, *P* = 0.016), lymph vessel invasion (OR 2.71, *P* = 0.026), T stage (OR 2.65, *P* = 0.037) and N stage (OR 2.34, *P* = 0.001) in resectable pancreatic cancer. Patients with positive peritoneal cytology demonstrated poor overall survival (OS; HR 3.18, *P* = 0.000) and disease-free survival (DFS; HR 2.88, *P* = 0.000) times. Based on our meta-analysis, we conclude that positive peritoneal cytology is an indicator of advanced stage pancreatic cancer with a poor prognosis; hence, radical resection should not be performed on these patients.

## INTRODUCTION

Pancreatic cancer is one of the leading causes of cancer-related mortality worldwide [1–3]. Although surgical resection is the only curable option, less than 20% of the patients diagnosed with localized disease are resectable. The prognosis of these patients after complete resection is extremely poor due to local and systemic recurrence [1, 4]. Since pre-operative imaging is inaccurate, re-staging the tumor based on laparoscopy, para-aortic lymph node sampling and intra-operative peritoneal cytology (CY) can rule out patients with occult tumor metastasis from unnecessary pancreatic resection [5–7].

Peritoneal cytology (CY) has been widely used in diagnosis and staging of ovarian, endometrial and gastric cancers [8–10]. In resectable pancreatic cancer patients, the incidence of positive peritoneal cytology (CY+) was 7–30% [11–20]. However, the prognostic significance of CY+ in potentially resectable pancreatic cancer is controversial. Some studies from Japan reported that CY+ without distant metastasis should not preclude resection in resectable pancreatic cancer patients and that long-term survival was possible after adjuvant chemotherapy [15, 16, 20]. In contrast, several other studies showed that patients with CY+ status were associated with advanced disease and poor prognosis and had survival rates equivalent to other stage IV diseases [11–14]. Due to these inconsistent conclusions, the American Joint Committee on Cancer and treatment guideline from the National Comprehensive Cancer Network considered CY+ as stage IV metastasis and contraindicator for pancreatic resection [21, 22]. However, the Pancreas Society in Japan did not include the CY status in stage evaluations to classify pancreatic cancer [23]. Therefore, in this study, we conducted this systemic review and meta-analysis to clarify the clinicopathological and prognostic significance of CY+ in resectable pancreatic cancer.

## RESULTS

### Search results and study characteristics

**Figure 1:**
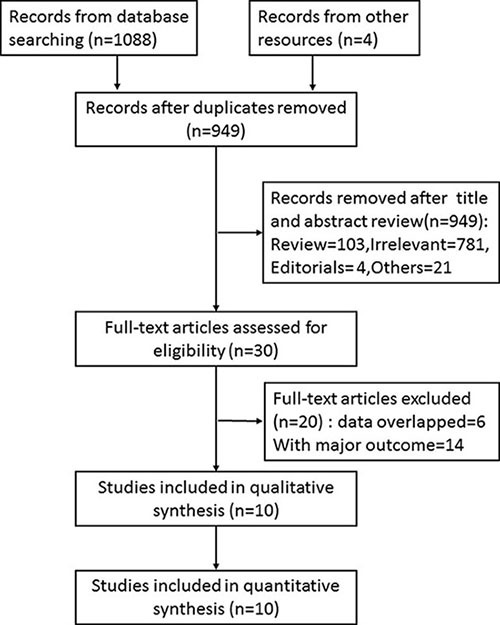
Flow chart showing selection criteria for including studies in the meta-analysis

Initially, 949 records were identified by searching the databases and other sources using the relevant terms as described in the methods section. After screening the title and abstract, 30 full-text articles were assessed for eligibility and 10 retrospective studies that met the inclusion criteria were included in this meta-analysis (Figure 1).

The characteristics of the included studies are summarized in Table 1. Of the 10 studies, 8 were from Japan and 2 from the USA. Combined, 228 CY+ and 2294 CY- patients were included in these studies and the incidence of CY+ was 11.8%. Papanicolaou and Giemsa staining were the most commonly used cytology methods. The follow-up time for the CY+ group ranged from 11 to 37 months with OS of 6–23.8 months, whereas, the CY- group had a OS of 16–36.4 months. Based on Newcastle-Ottawa quality assessment, three studies achieved 7 or more stars and were considered as high quality (Supplementary Table 1).

**Table 1:** Characteristis of studies included in the meta-analysis

Author	Year	Country	Method of cytology	No. of patients(CY+ vs CY-)	Median follow-up (months)	Median survival (months)	Adjuvant therapy	Multivariate/ Univariate	HR(95%CI)	Quality of study
Iwagami[11]	2015	Japan	Papanicolaou staining	5/34	31.0 (5.4-113.9)	OS: 13.3 vs 36.4 RFS: 6.5 vs 15.7	Gemcitabine based chemotherapy: 100% vs 55.9%	Multivariate	OS:2.589(0.734–8.532) RFS:5.100(1.279-20.925)	8
Satoi[12]	2015	Japan	Papanicolaou and/or Giemsa staining	69/915	19.7	OS: 16.0 vs 24.9 DFS: 8.8 vs 13.8	Gemcitabine or S-1 based chemotherapy: 86% vs 82%	Multivariate	OS:0.74 (0.55–0.98)^#^	6
Hirabayas hi [13]	2015	Japan	Papanicolaou and/or Giemsa staining	18/141	NA	OS: 10 vs 27	NA	Multivariate	OS:2.711(1.464-5.022)	6
Aoyama[14]	2015	Japan	Papanicolaou staining	21/122	37(4.4-110.5)	5 ys OS: 8.6% vs 16.1% 5 ys RFS: 0% vs 16.1%	Gemcitabine or S-1 based chemotherapy: 100%	Univariate	OS:1.370(0.796-2.361) RFS:2.360(1.427-3.904)	7
Yamada[15]	2013	Japan	Papanicolaou and Giemsa staining	51/339	15.7	OS: 14·3 vs 18·0	Gemcitabine or S-1 based chemotherapy: 53.3%	Multivariate	OS:1.36(0.94-1.90)^&^	6
Yoshioka[16]	2012	Japan	Papanicolaou staining	20/234	24.7(0.8-97.4)*	OS:23.8 vs 26.5 RFS: 8.1 vs 13.5	Chemotherapy: 80.0% vs 70.9%	NA	NA	8
Ferrone[17]	2006	US	NA	77/385^†^	11(0-122)	OS: 8 vs 16	In resected patients: 49%	NA	NA	6
Meszoely[18]	2004	US	H&E and Papanicolaou staining	27/141^‡^	NA	OS: 15 vs19 DFS:10 vs 12	5-FU based chemotherapy: 60% in resected tumors	NA	NA	6
Konishi[19]	2002	Japan	Papanicolaou staining	36/115^#^	NA	OS:NA	IORT(+ERBT+5-FU) in local advanced and metastatic tumor: 100%	NA	NA	6
Yachida[20]	2002	Japan	Papanicolaou, Giemsa and Alcian blue staining, ICH	16/114	NA	OS:18 vs 15	Mitomycin C or 5-FU based chemotherapy in 12 patients with R1 resection	NA	NA	6

* presented with mean (range). ^†^ there were 10 and 217 resected patients in Cy+ and Cy- groups, respectively. ^‡^ there were 13 and 122 resected patients in Cy+ and Cy- groups, respectively ^#^. there were 5 and 56 resected patients in Cy+ and Cy- groups, respectively. ^§^ presented with Cy- vs Cy+. ^&^ for resected tumors. NA not available.

### Meta-analysis results

### CY+ and clinicopathological characteristics

To explore the association between CY+ and clinicopathological features in resectable pancreatic cancer, binary meta-analysis was performed with ORs indicating outcomes. The results are summarized in Table 2. In brief, CY+ significantly correlated with tumor size (OR 95% CI, 11.65 (2.62–51.86.93); *P* = 0.001), tumor location (OR 95% CI, 0.37 (0.27–0.52); *P* = 0.000), serosal invasion (OR 95% CI, 3.89 (2.26–6.71); *P* = 0.000), portal vein invasion (OR 95% CI, 1.82(1.12–2.95); *P* = 0.016), lymph vessel invasion (OR 95% CI, 2.71 (1.13–6.50); *P* = 0.026), T stage (OR 95% CI, 2.65 (1.06–6.63); *P* = 0.037) and N stage (OR 95% CI, 2.34 (1.44–3.82); *P* = 0.001).

**Table 2:** Clinicopathological characteristics of CY+ and CY- patients with resectable pancreatic cancer

Outcome	Ref. included	No. of patients (CY+ vs CY-)	Heterogeneity test			Model used	OR	95%CI	*P* value
			Chi-square	*P* value	I-square				
Age (older vs younger)	[11, 13, 15]	74 vs 514	7.83	0.020	74.5%	Random model	2.40	0.38–15.38	0.354
Sex (male vs female)	[11–16, 18]	211 vs 1926	10.14	0.119	40.8%	Fixed model	0.96	0.71–1.29	0.762
Tumor size (larger vs smaller)	[11, 13, 15, 16]	94 vs 748	9.79	0.020	69.4%	Random model	11.65	2.62–51.86	0.001
Tumor location (head vs body/tail)	[11–15, 18]	177 vs 1673	6.60	0.169	39.4%	Fixed model	0.37	0.27–0.52	0.000
Tumor grade (poor vs well/moderate)	[11, 13, 14, 16]	64 vs 531	1.71	0.635	0%	Fixed model	0.89	0.40–1.97	0.776
Retroperitoneal invasion (Yes or No)	[11, 15, 16, 20]	92 vs 721	2.64	0.450	0%	Fixed model	1.63	0.97–2.72	0.063
Serosal invasion(yes or no)	[11, 15, 16, 20]	92 vs 721	4.27	0.234	29.7%	Fixed model	3.89	2.26–6.71	0.000
Portal vein invasion(yes or no)	[11, 15, 16]	76 vs 606	2.45	0.294	18.2%	Fixed model	1.82	1.12–2.95	0.016
Arterial invasion(yes or no)	[11, 15, 16]	76 vs 606	1.83	0.401	0%	Fixed model	1.85	0.86–3.96	0.115
Lymph vessel invasion(yes or no)	[11, 13, 14]	44 vs 297	0.34	0.842	0%	Fixed model	2.71	1.13–6.50	0.026
Perineural invasion(yes or no)	[11, 13, 15, 16]	94 vs 748	1.11	0.776	0%	Fixed model	0.93	0.53–1.61	0.785
Venous invasion(yes or no)	[11, 13, 14]	44 vs 297	2.51	0.285	20.3%	Fixed model	2.80	1.06–7.41	0.285
T stage(T3+4 vs T1+2)	[11–14, 19]	118 vs 1268	0.62	0.961	0%	Fixed model	2.65	1.06–6.63	0.037
N stage(N1 vs N0)	[11–14, 19]	118 vs 1268	6.33	0.176	36.8%	Fixed model	2.34	1.44–3.82	0.001

### CY+ and recurrence in resectable pancreatic cancer

**Figure 2:**
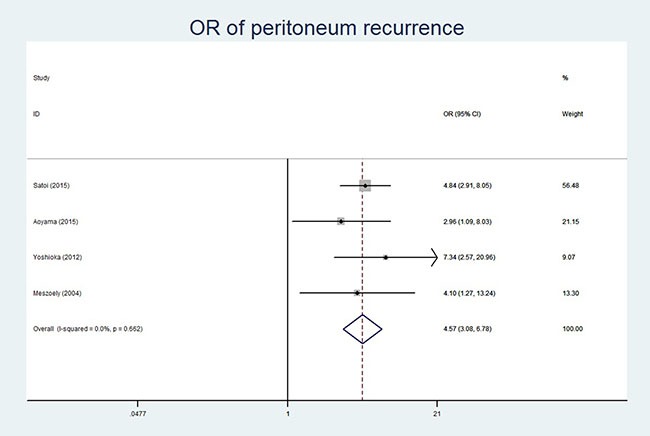
Forest plot of Odds ratio showing association of CY+ status and peritoneum recurrence

Four of the ten studies reported the relationship between CY+ and total recurrence [11, 12, 14, 18]. Since there was no significant heterogeneity (*P* = 0.205, I^2^ = 36.4%), the fixed-effect model was used for analysis. Our results revealed that CY+ was associated with total recurrence (OR 95% CI, 5.21 (2.45–11.00); *P* = 0.000). Further, we explored the correlation between the CY status and local recurrence or liver metastasis that was reported in four studies [12, 14, 16, 18]. Since no significant heterogeneity was detected (*P* = 0.983, I^2^ = 0% for local recurrence; *P* = 0.556, I^2^ = 0% for liver metastasis and *P* = 0.662, I^2^ = 0% for peritoneum recurrence), the fixed-effect model was used for meta-analyses. Our analysis detected no correlation between CY+ and local recurrence (OR 95% CI, 1.01 (0.60–1.70); *P* = 0.973) or liver metastasis (OR 95% CI, 0.75 (0.47–1.19); *P* = 0.214). However, CY+ status was associated with peritoneum recurrence (OR 95% CI, 4.57 (3.08–6.78); *P* = 0.000; Table 3, Figure 2).

**Table 3:** Meta-analysis results for OS, DFS and peritoneum recurrence

Outcome	Ref. included	No. of patients (CY+ vs CY−)	Heterogeneity test			Model used	HR	95%CI	*P* value
			Chi-square	*P* value	I-square				
OS	[11–20]	228 vs 2294	85.08	0.000	89.4%	Random model	3.18	1.88–5.39	0.000
Quality of research									
High	[11, 14, 16]	46 vs 390	5.77	0.056	65.3%	Random model	2.31	1.11–4.83	0.026
Low	[12, 13, 15, 17–20]	182 vs 1904	56.60	0.000	89.2%	Random model	4.06	2.00–8.24	0.000
Origin of research									
Japan	[11–16, 19, 20]	205 vs 1955	41.57	0.000	83.2%	Random model	2.42	1.53–3.82	0.000
US	[17, 18]	23 vs 339	3.12	0.077	68.0%	Random model	9.07	3.98–20.69	0.000
Adjuvant chemotherapy									
Gemcitabine based	[11, 12, 14, 15]	146 vs 1410	1.04	0.792	0%	Fixed model	1.38	1.13–1.69	0.002
Others	[13, 16–20]	82 vs 884	16.18	0.006	69.1%	Random model	5.28	3.28–8.49	0.000
DFS	[11,12, 14, 16, 18]	128 vs 1427	4.86	0.302	17.7%	Fixed model	2.88	2.39–3.49	0.000
Quality of research									
High	[11, 14, 16]	46 vs 390	2.40	0.301	16.7%	Fixed model	3.09	2.16–4.44	0.000
Low	[12, 18]	82 vs 1037	2.26	0.133	55.7%	Random model	3.25	1.84–5.76	0.000
Origin of research									
Japan	[11, 12, 14, 16]	115 vs 1305	2.86	0.414	0%	Fixed model	2.79	2.29–3.39	0.000
US	[18]	13 vs 122	NA	NA	NA	Fixed model	5.00	2.28–10.98	0.000
Adjuvant chemotherapy									
Gemcitabine based	[11, 12, 14]	95 vs 1071	1.05	0.591	0%	Fixed model	2.65	2.15– 3.26	0.000
Others		33 vs 356	0.21	0.646	0%	Fixed model	4.31	2.73–6.79	0.000
Peritoneum recurrence	[12, 14, 16, 18]	123 vs 1393	1.59	0.662	0%	Fixed model	4.57	3.08–6.78	0.000
Quality of research	[16, 18]								
High	[14, 16]	41 vs 356	1.54	0.215	35%	Fixed model	4.27	2.06–8.86	0.000
Low	[12, 18]	82 vs 1037	0.06	0.800	0%	Fixed model	4.70	2.94–7.50	0.000
Origin of research									
Japan	[12, 14, 16]	110 vs 1271	1.54	0.464	0%	Fixed model	4.64	3.06–7.05	0.000
US	[18]	13 vs 122	NA	NA	NA	Fixed model	4.10	1.27–13.24	0.018
Adjuvant chemotherapy									
Gemcitabine based	[12, 14]	90 vs 1037	0.74	0.389	0%	Fixed model	4.33	2.75–6.81	0.000
Others	[16, 18]	33 vs 356	0.54	0.464	0%	Fixed model	5.42	2.44–12.00	0.000

### CY+ and OS in resectable pancreatic cancer

**Figure 3:**
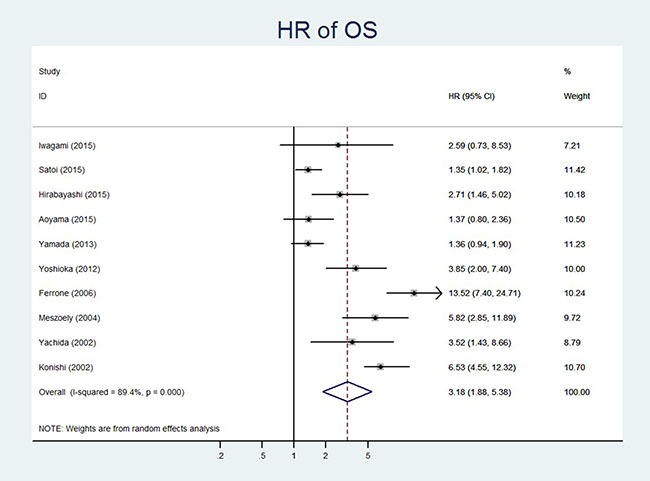
Forest plot of hazard ratio showing association of CY+ status and OS

All the 10 included studies reported the OS data with 228 and 2294 patients in CY+ and CY- groups, respectively [11–20]. Since the data were heterogeneous (*P* = 0.000, I^2^ = 89.4%), a random-effect model was used. The pooled HR for OS showed that CY+ in resectable pancreatic cancer was associated with poor OS (HR 95%CI, 3.18(1.88–5.39; *P* = 0.000) (Table 3, Figure 3).

### CY+ and DFS in resectable pancreatic cancer

**Figure 4:**
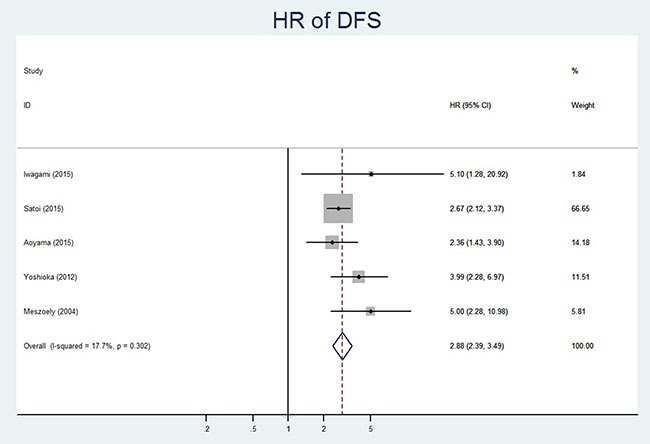
Forest plot of hazard ratio showing association of CY+ status and DFS

Five of the ten studies reported the DFS data with 128 CY+ patients and 1427 CY- patients [11, 12, 14, 16, 18]. The fixed effect model was used for analysis as significant heterogeneity was not detected (*P* = 0.302, I^2^ = 17.7%). Our analysis demonstrated that CY+ status was associated with poor DFS (HR 95%CI, 2.88(2.39–3.49); *P* = 0.000) in resectable pancreatic cancer patients (Table 3, Figure 4). The results of the subgroup analysis were consistent with the overall analysis and are summarized in Table 3.

### Publication bias

**Figure 5:**
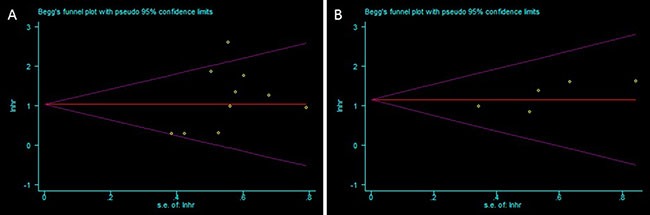
Funnel plots analyzing publication bias in this meta-analysis for (**A**) Overall survival and (**B**) Disease-free survival.

Begg's test showed no publication bias in the studies used for the meta-analysis with OS (*P* = 0.142) and DFS (*P* = 0.129) as shown in Figure 5.

## DISCUSSION

The increased incidence of CY+ from 7–30% in resectable to 20–57% in unresectable pancreatic cancer [11–20, 24] suggested that CY+ patients had greater tumor burden and represented a more advanced stage of cancer. Previously, CY+ was associated with high pre-operative CA19-9 levels [12], larger tumors [12, 13, 15, 20, 25], tumor location in the body/tail of the pancreas [12, 13, 26], lymph node metastasis [12], vascular invasion [11], serosal invasion [15, 16] and late TNM stage [13, 26]. Our results showed that CY+ was more frequent in advanced resectable pancreatic cancer patients and was significantly associated with larger tumor size (OR 11.65, *P* = 0.001), tumor located in pancreatic head (OR 0.37, *P* = 0.000), serosal invasion (OR 3.89, *P* = 0.000), portal vein invasion (OR 1.82, *P* = 0.016), lymph vessel invasion (OR 2.71, *P* = 0.026), T stage (OR 2.65, *P* = 0.037) and N stage (OR 2.34, *P* = 0.001).

The prognosis of pancreatic cancer with synchronous peritoneal metastasis was extremely poor with a median survival of 6 weeks (95% CI, 5–7weeks) and no improvement inspite of chemotherapy [27]. In the gastric, colon and gynecological malignancies, microscopic occult peritoneal metastases preceded the emergence of macroscopic metastases and such patients were precluded from radical surgery [8, 28–30]. However, the impact of CY+ on long-term survival of pancreatic cancer was uncertain. Some surgeons from Japan believed that CY+ status in the absence of other distant metastasis was not a contraindication for radical surgery and that surgical resection offered a chance for long-term survival [15, 16, 20, 25]. In contrast, other studies suggested that patients with CY+ experienced peritoneal recurrence and poor survival time inspite of radical surgery and adjuvant chemotherapy [11–14]. In a recent large-scale multicenter study with 984 cases receiving R0 resection, Satoi and colleagues reported that OS of CY+ patients (median survival time: 16 months; 3 year OS rate: 6 %) was worse than CY− patients (median survival time: 25 months; 3-year OS rate: 37 %; *p* < 0.001). Also, CY+ patients demonstrated higher post-operative peritoneal carcinomatosis (48%) than the CY− patients (21%; *p* < 0.001) [12]. Our data revealed that CY+ status was a risk factor for poor OS (HR 3.18, *P* = 0.000), DFS (HR 2.88, *P* = 0.000) with high total recurrence (HR 5.21, *P* = 0.000) and peritoneal recurrence (HR 4.57, *P* = 0.000) in patients with resectable tumor. Although some surgeons from Japan performed more aggressive resection for pancreatic cancer than those from US, the OS of Japan patients was similar with US patients. Our subgroup analysis still revealed that CY+ was a predictor of shorter OS and DFS with a higher recurrence rate in Japan patients.

The median OS time in resectable CY+ patients ranged from 8–23.8 months [16, 17] that was better than the 6.7–17 months range for the metastatic pancreatic cancer patients receiving either gemcitabine, nab-paclitaxel plus gemcitabine or Folfirinox chemotherapy [31–33]. However, the prognosis of CY+ patients who underwent pancreatic resection was poorer than the locally advanced tumor patients receiving aggressive modern chemotherapy without surgery with a median OS time was 10.0–32.7 months [34, 35]. Also, CY+ patients failed to display long-term DFS upon resection of the primary tumor. Therefore, CY+ was equivalent to M1 disease and radical surgery was not advisable.

Since unnecessary pancreatic resection could delay the induction of chemotherapy that would adversely affect patient survival [34]. In a recently updated Cochrane systemic review, prior diagnostic laparoscopy with biopsy and histopathological confirmation of suspicious lesions resulted in avoiding 21% unnecessary laparotomies planned with a curative intent for cancer resection [35]. Similarly, we recommend the routine use of staging laparoscopy and peritoneal washing cytology examination in patients with resectable pancreatic cancer to detect occult tumors and avoid unnecessary surgery.

When intra-operative cytology results are unavailable, we propose that patients receive adjuvant chemotherapy as soon as CY+ status is confirmed. In cases where CY+ status is observed during operation, intra-operative HIPEC (hyperthermic intraperitoneal chemotherapy) followed by adjuvant chemotherapy should be considered to effectively control the local recurrences [36, 37]. Also, the CY+ patients should be treated as M1 postoperatively regardless of status of primary tumor.

Although molecular targeted therapy has been extensively evaluated, so far they have been ineffective in pancreatic cancer. EGFR inhibition using erlotinib is the only trial that demonstrated improved survival [38]. Pancreatic cancer has been refractory to immunotherapy through CTLA-4, PD-1 or PD-L1 antibodies that have been promising for many advanced solid tumors [39, 40]. Data from several new agents is currently under investigation and the results are expected in the next year or two.

In unresected CY+ tumors without other macrometastasis, re-staging laparoscopy should be considered in patients showing favorable response to chemotherapy. If CY- status is confirmed, pancreatic resection can be performed. Recent studies showed that resection of the primary tumor in metastatic pancreatic adenocarcinoma patients following favorable response to systemic chemotherapy resulted in improved survival [41, 42].

This meta-analysis had several limitations. First, significant heterogeneity was observed among the included studies regarding OS analysis. Although the random-effect models were used to pool the OS data, the heterogeneity may have reduced the effect of large-sample studies of good quality. The possible reasons for heterogeneity included the method for cytology, small number of included studies and the statistical approach for extrapolating HRs. Second, the imprecise estimation of HRs from the Kaplan-Meier curves could have adversely influenced the conclusions from our meta-analysis. Third, since all the included studies were retrospective, bias regarding selection, information and other parameters need to be considered. Inspite of these shortfalls, our results were reliable because no publication bias was detected and the subgroup analyses results were similar.

In conclusion, CY+ status was associated with advanced tumor and poor prognosis, radical resection should not be performed on such pancreatic cancer patients.

## MATERIAL AND METHODS

### Publications search and Selection criteria

This meta-analysis was conducted in accordance with the PRISMA (Preferred Reporting Items for Systematic Reviews and Meta-Analyses) guidelines. A computerized search was performed for the terms “pancreatic cancer or pancreatic adenocarcinoma” and “peritoneal cytology” by searching PubMed, Web of Science and China National Knowledge Infrastructure(CNKI) databases in August 2016. No language restrictions were applied. The reference list in the selected articles was also checked. The criteria for including studies in the meta-analysis were: (a) clinical studies researched patients with potentially resectable pancreatic cancer; (b) CY status was measured; (c) at least one of the three endpoints (overall survival(OS), Disease-free survival(DFS) or recurrence) were reported and (d) studies contained a hazard ratio (HR) or odds ratio (OR) with the corresponding confidence interval (CI) or sufficient data to calculate them. The criteria to exclude articles from the analysis were: (a) letters, case reports, reviews and conference abstracts without original data; (b) duplicates of previous publications and (c) articles without key information such as Kaplan-Meier curves, HRs with 95% CIs or clinicopathological features.

### Data extraction

Two reviewers (F. Cao and J. Li) independently considered the eligibility of potential titles and extracted the following information: first author's surname, year of publication, number of patients, method of cytology, median time of follow-up and survival, regimen of adjuvant therapy, recurrence rate, HR with 95% CI, patient age and sex, tumor location, size, tumor grade and progression, T and N stage. Discrepancies were resolved by mutual discussion or consulting with the third reviewer (A. Li).

### Quality assessment

The Newcastle-Ottawa Scale was used to assess the quality of the included studies [43]. According to this scale, the maximum score could be nine points that indicated highest methodological quality. NOS score of 7 or above was considered as high quality whereas a NOS score of 3 or below was considered low quality.

### Main outcomes

The primary outcomes of this study were the prognostic significance (recurrence, OS and DFS) of CY+ in potentially resectable pancreatic cancer. The secondary outcomes included calculating the Odds ratios (ORs) of clinicopathological features for CY+ versus CY- patients.

### Statistical analysis

HRs and 95% CIs were used to measure the effective value. We used the HRs that was already calculated in the published studies, whenever available. If not, we calculated the HR and 95% CIs from the Kaplan-Meier survival curve or other relevant data using methods reported by Tierney and colleagues [44]. Data from the Kaplan-Meier survival curves were read using the Engauge Digitizer version 4.1. A combined HR/OR > 1 indicated poor outcome for CY+ patients. The chi-square *Q* test and I^2^ statistics were used to explore the heterogeneity. If heterogeneity was significant (*P* < 0.1 or I^2^ > 50%), the M-H or I-V heterogeneity model was used. Otherwise, a fixed-effects model of Mantel-Haenszel was applied. Subgroup analyses for primary outcomes were performed if necessary data was available. Publication bias was analyzed using the Egger's test. All the statistical analyses were performed with the STATA/SE software version12.0 (STATA Corporation, College Station, TX, USA).

## SUPPLEMENTARY MATERIALS FIGURES AND TABLES


