# Supplementary material for: Prognostic significance of positive peritoneal cytology in resectable pancreatic cancer: a systemic review and meta-analysis
Source: Oncotarget. 2017 Jan 19;8(9):15004–13. doi: 10.18632/oncotarget.14745 (PMC5362462; doi:10.18632/oncotarget.14745)
Supplement: Supplementary file 1 [file oncotarget-08-15004-s001.pdf]

Prognostic significance of positive peritoneal cytology in resectable pancreatic cancer: a systemic review and meta-analysis

Supplementary Materials

Supplementary Table 1: Risk of bias in the included retrospective cohort studies (by the Newcastle–Ottawa quality assessment tool)

References	Selection/4	Comparability/2	Outcome/3	Total score
Iwagami	3	2	3	8
Satoi	3	1	2	6
Hirabayashi	3	1	2	6
Aoyama	3	1	3	7
Yamada	3	1	2	6
Yoshioka	3	2	3	8
Ferrione	3	1	2	6
Meszoely	3	1	2	6
Konishi	3	1	2	6
Yachida	3	1	2	6

Appendix: Newcastle-Ottawa Scale (NOS) for quality assessment

**Selection**	
Representativeness of the exposed cohort	a) truly representative of the average potentially resectable pancreatic cancer in the community (※)<br>b) somewhat representative of the average potentially resectable pancreatic cancer in the community (※)<br>c) selected group of users eg nurses, volunteers<br>d) no description of the derivation of the cohort
Selection of the non-exposed cohort	a) drawn from the same community as the exposed cohort (※)<br>b) drawn from a different source<br>c) no description of the derivation of the non- exposed cohort
Ascertainment of exposure	a) surgical records (※)<br>b) structured interview (※)<br>c) written self report<br>d) no description
Demonstration that outcome of interest was not present at start of study	a) yes (※)<br>b) no
**Comparability**	
Comparability of cohorts on the basis of the design or analysis	a) The tumor size between exposed cohort and non-exposed cohort had no statistical differences(※)<br>b) The age(sex, T, N stage etc) between exposed cohort and non-exposed cohort had no statistical differences (※)
**Outcome**	
Assessment of outcome	a) independent blind assessment (※)<br>b) record linkage (※)<br>c) self report<br>d) no description
Was follow-up long enough for outcomes to occur? (death or recurrence)	a) yes (24 months) (※)<br>b) no
Adequacy of follow up of cohorts	a) complete follow up - all subjects accounted for (※)<br>b) subjects lost to follow up unlikely to introduce bias - small number lost (less than 25%) or description provided of those lost) (※)<br>c) follow up rate < 75% and no description of those lost<br>d) no statement

Note: A study can be awarded a maximum of one star for each numbered item within the Selection and Outcome categories. A maximum of two stars can be given for Comparability.
